# Right broca homologue mediates the pain-depression circuit: a case-control Functional Near-Infrared Spectroscopy (fNIRS) study on language network remodeling in chronic pain-depression comorbidity*

**DOI:** 10.1186/s12888-026-08323-3

**Published:** 2026-06-23

**Authors:** Jiaren Zheng, Ning Zhu, Jiaxi Huang, Hongyu Sun, Huimin Lv, Jing Zhang, Zhenhua Li, Manyu Zhang, Jing Li, Bo Zheng, Yibin Xie, Zhong Zheng

**Affiliations:** 1 Xiamen Key Lab of Psychoradiology and Neuromodulation, Function Testing and Modulation Center for Neurological, West China Hospital, West China Xiamen Hospital, Sichuan University, Xiamen Fujian, 361000 China; 2 The Third Hospital of Jinjiang, Quanzhou, 362211 China; 3 West China Hospital of Sichuan University, Chengdu, 610041 China; 4 Xiamen Xianyue Hospital, Xiamen, 361012 China

**Keywords:** Major depressive disorder, Chronic pain, fNIRS, Broca's homologue, Neuroplasticity, Mediation analysis, Prefrontal cortex

## Abstract

**Background:**

Chronic somatic pain (CSP) and major depressive disorder (MDD) frequently co-occur and are associated with poor prognosis. However, the neural circuit mechanisms underlying the modulation of MDD pathology by CSP remain unclear.

**Methods:**

This case-control study included 129 participants who underwent fNIRS during a verbal fluency task (VFT) and at rest. Participants were divided into three groups (*n* = 43 each): MDD patients with comorbid CSP (MDD + CSP), MDD patients without CSP (MDD-CSP), and healthy controls (HC). This study aimed to identify CSP-specific neural signatures and examine their mediating role between pain and depression.

**Results:**

Compared with HC, all MDD patients exhibited reduced activation in the bilateral frontopolar cortex during the task. A key finding was that, relative to both HC and the MDD-only group, the comorbid group showed selectively lower activation in the right-hemispheric Broca’s homologue (RH-Broca) (*p* = 0.003). Pain intensity was negatively correlated with RH-Broca activation (*p* < 0.001). Mediation analysis revealed a bidirectional mediating effect of RH-Broca activation on the pain-depression relationship: it mediated both the pathway from pain to depression (indirect effect *β* = 1.17, 95% CI: 0.66–1.71) and the pathway from depression to pain (indirect effect *β* = 0.13, 95% CI: 0.08–0.18). At rest, the comorbid group displayed hyperactivation in the right frontopolar cortex, whereas the MDD-only group showed alterations in small-world network properties.

**Conclusion:**

Selective hypoactivation in the right-hemispheric language network, particularly in the RH-Broca, is closely associated with the comorbidity of chronic somatic pain and major depressive disorder and may contribute to their mutual exacerbation. These findings support the view that the RH-Broca serves as a putative neural node connecting pain and depressive symptoms, potentially forming a bidirectional loop in comorbid patients. This neural circuit may represent a promising target for neuromodulation therapy in patients with MDD comorbid with CSP.

**Supplementary Information:**

The online version contains supplementary material available at https://doi.org/10.1186/s12888-026-08323-3.

## Background

Major Depressive Disorder (MDD) is a psychiatric condition characterized by persistent low mood, diminished interest, and anhedonia. It is frequently accompanied by significant cognitive impairment and somatic symptoms, such as chronic pain (CP) [1, 2]. MDD exhibits high prevalence, recurrence rates, and disability burden [3–5]. Globally, the number of individuals affected exceeds 1 billion, with a trend toward earlier onset [6, 7]. In China, the lifetime prevalence of MDD is 6.8% [6, 8], and approximately 66%–75% of MDD patients report comorbid chronic or unexplained somatic pain [9]. This comorbidity of depression and Chronic Somatic Pain (CSP) not only complicates clinical diagnosis and treatment [10–12] but also leads to greater social functional disability and a heavier healthcare burden [13, 14]. Growing evidence suggests that the clinical presentation of comorbid depression and CSP is far more complex than a simple superposition of two independent disorders [15–17].

Recent research has moved beyond traditional frameworks of peripheral and central sensitization, increasingly focusing on the potential systematic remodeling of advanced brain circuits [18] It is widely accepted that these two conditions share critical neurobiological substrates, including neuroimmune inflammation and dysregulation of reward circuits, which constitute a common foundation for comorbidity [18, 19]. However, emerging evidence further reveals fine-grained functional segregation within these shared circuits. For instance, within the nucleus accumbens—a key hub for reward and motivation integration—neurons expressing D1 versus D2 dopamine receptors have been shown to independently regulate depressive-like behaviors and pain sensitization through distinct downstream pathways [20]. This indicates that the comorbid state is a complex pathological phenomenon, intertwined at the systems level yet specified at the cellular and circuit levels. The interactive mechanisms specifically involve: (1) shared molecular pathways for “pain-negative emotion,” such as downregulated gastrin-releasing peptide signaling in the medial orbitofrontal cortex to nucleus accumbens pathway [21]; (2) a behavioral vicious cycle of “pain-reduced activity-depression” [16]; and (3) key processes like sustained neuroimmune activation mediated by glial cells [18]. Despite significant progress in mechanistic understanding, translating insights regarding circuit-level “sharing” and “separation” into effective treatment strategies that simultaneously target both symptom domains remains a core challenge. Therefore, elucidating these circuit-level interactions requires a research tool capable of probing cortical activity with high ecological validity under natural task conditions.

fNIRS offers a unique technical approach due to its non-invasive nature, tolerance to movement artifacts, and applicability in ecologically valid settings during complex cognitive tasks [22]. Based on the neurovascular coupling principle, fNIRS detects changes in cortical hemoglobin concentration to reflect neural activity [23]. In clinical research, fNIRS is often combined with cognitive paradigms such as the VFT, which has been widely validated to effectively engage prefrontal and language-related cortical areas [24], serving as a robust tool for assessing cognitive processing states [25]. In the field of depression, numerous fNIRS studies have confirmed characteristic abnormalities in prefrontal (particularly dorsolateral prefrontal) activation during VFT performance in patients, suggesting these may serve as potential objective biomarkers [26–30]. However, the application of fNIRS in the highly disabling clinical entity of MDD comorbid with CSP remains limited. Although preliminary evidence suggests an association between somatosensory cortex activity and emotional symptoms in chronic pain patients [31], and higher-order neural circuit dysfunctions involving reward, salience, and sensorimotor networks are considered central to comorbidity [32], direct fNIRS evidence is lacking for the crucial question of how CSP specifically modulates depression-relevant neural circuits, such as prefrontal-limbic interactions.

To address this research gap, this study employs a three-group case-control design, combining fNIRS with the VFT paradigm. The aims are: [1] to systematically compare differences in prefrontal neural activity and brain network characteristics during task and resting states among MDD patients with CSP, MDD patients without CSP, and healthy controls (HC); and [2] to investigate the mediating role of these neural indicators between pain intensity and depressive symptoms. Based on theories that pain may affect cognitive resource allocation and hemispheric lateralization of brain function, we hypothesize that CSP induces specific alterations in neural activity within the right hemisphere (involving the non-dominant hemisphere language network) in MDD patients, and that abnormalities in this region may play a key mediating role in the interaction between pain and depression. This study is expected to provide new neuroimaging evidence for understanding the comorbidity mechanism of MDD and CSP, and to suggest potential biomarkers for future targeted neuromodulation therapies (e.g., repetitive transcranial magnetic stimulation) aimed at specific circuits.

## Materials and methods

### Ethics statement

This study was approved by the Biomedical Huali Review Committee of West China Xiamen Hospital, Sichuan University (Approval No. [2024] Review (004)) and was conducted in accordance with the principles of the Declaration of Helsinki. The study was registered with the Chinese Clinical Trial Registry (Registration No.: ChiCTR2500098790). Written informed consent was obtained from all participants or their legal guardians before the commencement of the study.

### Study design and reporting guidelines

This was a case-control study conducted from December 2023 to March 2024 at the Neuropsychiatric Function Assessment and Modulation Center / Center for Mental and Neurological Disorders, West China Xiamen Hospital, Sichuan University. It aimed to investigate the neural correlates of Major Depressive Disorder (MDD) with or without comorbid Chronic Somatic Pain (CSP). The design, implementation, and reporting of this study strictly adhered to the Strengthening the Reporting of Observational Studies in Epidemiology (STROBE) statement for case-control studies [33]. A completed STROBE checklist is provided in Supplementary File 1.

### Participants

#### Sample size estimation

A priori sample size estimation was performed using G*Power 3.1 software. Based on previous fNIRS studies employing the VFT, which have consistently demonstrated medium-to-large effect sizes in prefrontal activation differences between individuals with major depressive disorder (MDD) and healthy controls [26, 27], we assumed an effect size *f* of 0.35 for *analysis of variance* (ANOVA) across three groups. To achieve 80% power at a two-tailed *a* level of 0.05, a minimum of 36 participants per group was required. Considering potential data attrition and to ensure robust statistical power for planned mediation analyses—which often require larger samples—we aimed to recruit 43 participants per group. This yielded a total target sample size of 129, which aligns with or exceeds the sample sizes of comparable case-control fNIRS studies in depression research [2, 29]. The reporting of this estimation adheres to the Strengthening the Reporting of Observational Studies in Epidemiology (STROBE) guidelines [33].

### Participants and grouping

A total of 186 potential participants were consecutively recruited and underwent eligibility assessment. Of these, 57 individuals were excluded for the following reasons: failure to meet the inclusion criteria (*n* = 27), refusal to participate (*n* = 13), and other causes (*n* = 17). Individuals with significant physical illnesses, substance abuse history, or personal/family history of psychiatric disorders were also excluded. Ultimately, 129 eligible participants were enrolled in the study and stratified into three well-matched groups (*n* = 43 per group) using a case-control design.

Participants were assigned to one of three cohorts as follows: Major depressive disorder with chronic somatic pain group (MDD + CSP, *n* = 43): Diagnosed with MDD and reporting chronic somatic pain persisting or recurring for ≥ 3 months, with a visual analog scale (VAS) score ≥ 4 [34]. Major depressive disorder without chronic somatic pain group (MDD-CSP, *n* = 43): Diagnosed with MDD, with a VAS score < 4. Healthy control group (HC, *n* = 43): Volunteers with no history of mental disorders, confirmed via the Structured Clinical Interview for DSM-IV Axis I Disorders-Non-Patient Edition (SCID-I/NP).

All MDD diagnoses in the case groups were independently confirmed by at least two attending psychiatrists at the center to ensure diagnostic accuracy. As an exploratory study, a convenience sample was used; this sample size (*n* = 129) is comparable to or larger than previous fNIRS studies with similar designs, and was deemed sufficient for preliminary group comparisons and mediation analyses. The three groups were matched for age and sex to ensure baseline homogeneity.

**Fig. 1:**
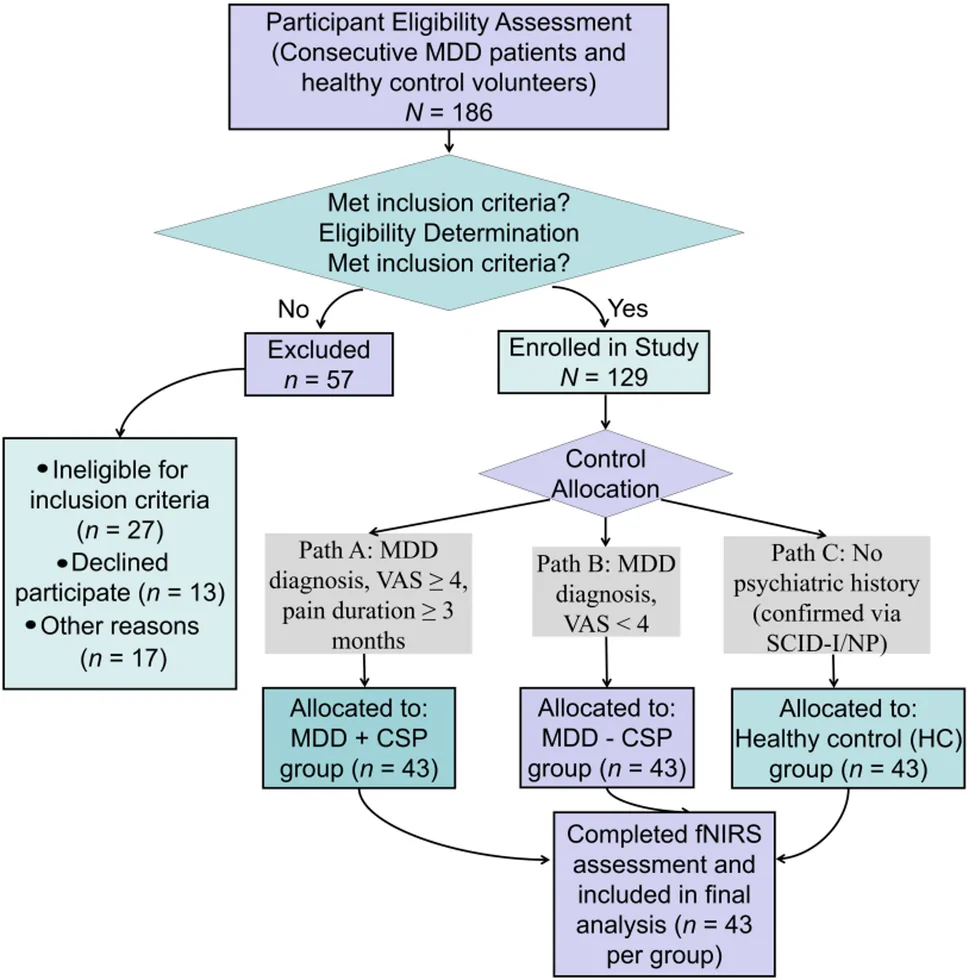
Flow diagram of participant recruitment, eligibility screening, and group allocation. Abbreviations: MDD, major depressive disorder; CSP, chronic somatic pain; VAS, visual analog scale; SCID-I/NP, Structured Clinical Interview for DSM-IV Axis I Disorders Non-Patient Edition; fNIRS, functional near-infrared spectroscopy

All enrolled participants completed fNIRS data acquisition, encompassing both a verbal fluency task and resting-state scanning. All datasets were subsequently subjected to regional activation analysis, functional connectivity analysis, mediation analysis, and correlation analysis. The participant flow is illustrated in Fig. 1.

### Inclusion and exclusion criteria

Inclusion criteria for the case groups: ① Diagnosis of MDD according to DSM-5 criteria; ② Education level > 6 years; ③ No significant visual or hearing impairment, with intact assessment capacity; ④ Agreement to participate and provision of signed informed consent.

Exclusion criteria (applied to all participants): ① Secondary mental disorders due to neurological or general medical conditions; ② Comorbid bipolar disorder, schizophrenia, or other severe psychiatric disorders; ③ History of severe failure of vital organs (heart, lungs, liver, kidneys, etc.); ④ Uncontrolled hypertension, arrhythmia, severe coronary heart disease, or poorly controlled diabetic complications; ⑤ Electroconvulsive Therapy (ECT) within the past 6 months; ⑥ Use of antibiotics/hormones/psychotropic medications within the past 30 days; ⑦ Pregnant or lactating women; ⑧ Poor compliance preventing cooperation with the study.

### Clinical assessments

#### Basic information collection

Demographic and clinical data were collected, including sex (biological), age, illness duration (for patients), as well as the nature and impact of somatic pain.

### Symptom assessment

Depressive Symptoms: Assessed using the Self-rating Depression Scale (SDS) [35]. The SDS consists of 20 items scored from 1 to 4. A standard score cut-off of 50 was applied: 50–59 indicates mild depression, 60–69 moderate depression, and ≥ 70 severe depression.

Pain Symptoms: Assessed using the Visual Analog Scale (VAS) [36]. The VAS is a measurement tool consisting of a 10 cm horizontal line where 0 indicates “no pain” and 10 indicates “worst possible pain.” Scores of 1–3, 4–6, and 7–10 indicate mild, moderate, and severe pain, respectively. Participants marked their perceived pain intensity on the line.

CSP was defined based on duration and intensity in this study, without distinguishing pain subtypes (e.g., neuropathic vs. nociceptive). This heterogeneity is acknowledged as a potential limitation in interpreting neural mechanisms.

### Functional near-infrared spectroscopy (fNIRS)

#### Experimental procedure

Participants were seated comfortably in a temperature-controlled (25 ± 1 °C), appropriately lit assessment room for 5 min to acclimate. They then wore the fNIRS head cap. Under quiet conditions, participants were instructed to close their eyes, relax, avoid limb movement, and listen attentively to and strictly follow the system’s audio prompts. To prevent habituation, each trial was performed only once.

#### Experimental paradigm

**Fig. 2:**
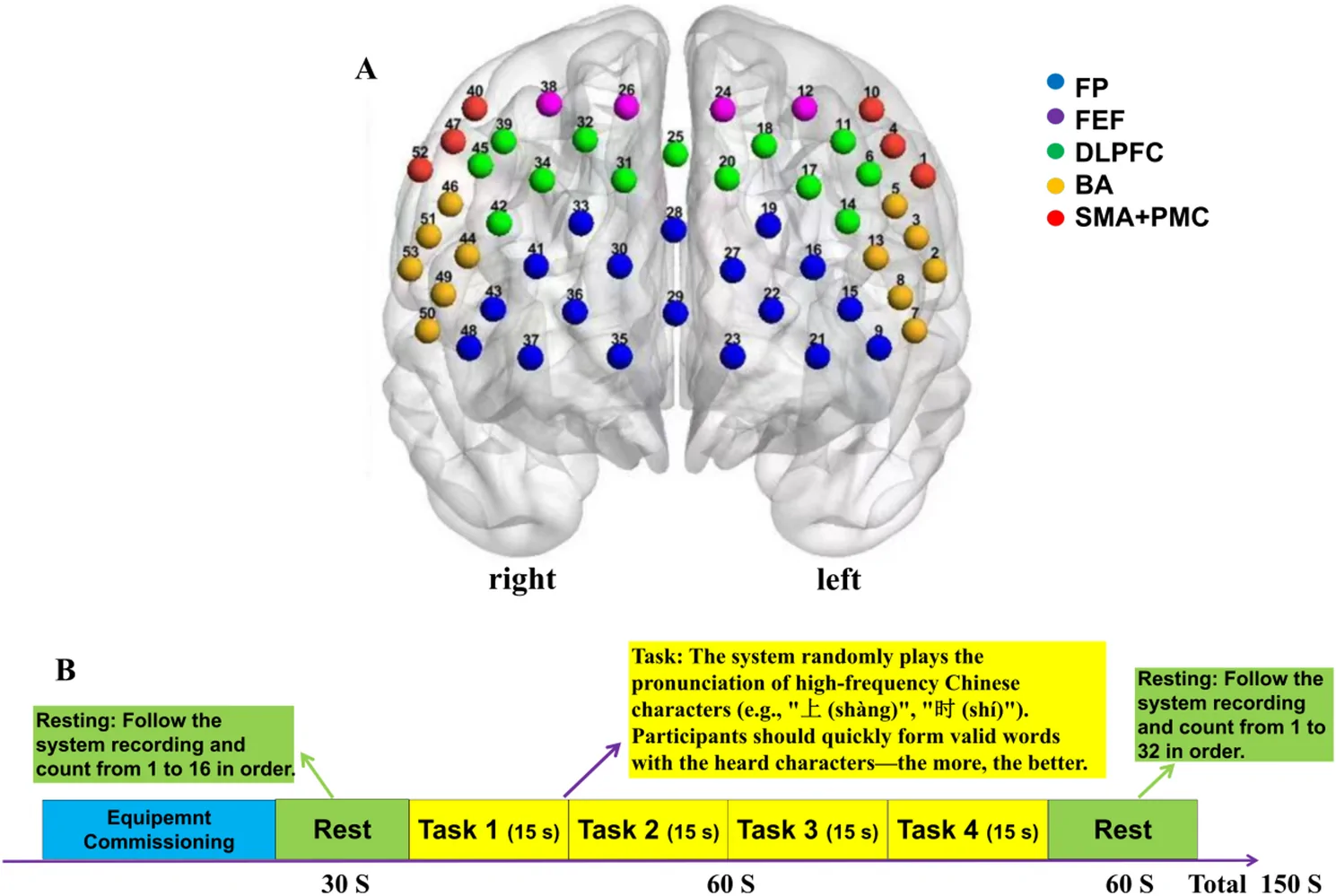
fNIRS channel configuration and Verbal Fluency Task (VFT) paradigm. (**A**) A 16-source, 16-detector optode array with 3 cm source-detector separation, forming 53 channels, covering the prefrontal and language-related cortices (FP: frontal pole; FEF: frontal eye fields; DLPFC: dorsolateral prefrontal cortex; BA: Broca’s area; SMA + PMC: supplementary motor area + premotor cortex). (**B**) A 150-second block design paradigm: 30-second baseline rest (counting 1–16), 60-second task period (generating words from high-frequency Chinese characters), and 60-second post-task recovery (counting 1–32). *n* = 43 per group

A VFT paradigm was employed, lasting 150 s in total, divided into three phases: (1) A 30-second baseline resting period: participants closed their eyes, relaxed, and silently counted from 1 to 16 following system prompts to maintain alertness. (2) A 60-second task period: the system randomly presented auditory cues of four high-frequency Chinese characters (e.g., “上/shàng”, “时/shí”, “说/shuō”, “家/jiā”). Following each cue, participants were required to generate as many non-repeated words related to the target character as possible within 15 s. (3) A 60-second post-task resting period: participants closed their eyes and relaxed again, silently counting from 1 to 32 following prompts. A schematic of the paradigm is shown in Fig. 2.

### Data acquisition

All participants underwent fNIRS acquisition using the identical device, task paradigm, and assessment protocol to ensure comparability across groups. Specifically, data were acquired using a BS-3000 fNIRS system (Zilian Hongkang, China) with dual wavelengths (690 nm/830 nm) at a sampling rate of 20 Hz. The probe array consisted of 16 light sources and 16 detectors (3 cm separation), forming 53 channels. Based on the EEG-10-20 system landmarks (F3/F4/Fz), channel positions were registered to MNI space using NIRSite software [37], covering the premotor and supplementary motor cortex, Broca’s area, and dorsolateral prefrontal cortex. Oxyhemoglobin (HbO) concentration changes were calculated using the modified Beer-Lambert law [38] (DPF₆₉₀ = 5.8, DPF₈₃₀ = 4.9). Average HbO concentrations (Avg-HbO) were extracted for the task period (35–90 s) and resting periods (5–30 s, 95–150 s).

### fNIRS data preprocessing

Raw light intensity signals were processed using the Homer2 toolbox [39] based on the MATLAB platform (R2013b, MathWorks Inc.) following these steps: (1) Signal Quality Check & Bad Channel Removal: The standard deviation of raw light intensity for each channel was calculated. Channels with a coefficient of variation > 6% or peak HbO concentration change > 0.5 µM were marked as bad and excluded from subsequent analysis. (2) Filtering & Denoising: A 4th-order Butterworth bandpass filter (0.008–0.2 Hz) was applied to remove physiological noise (e.g., respiration, heart rate) and slow drifts. Subsequently, Principal Component Analysis was used to remove the first principal component accounting for > 95% of signal variance, effectively suppressing motion artifacts and other global interferences [40]. (3) Hemoglobin Concentration Conversion: Preprocessed light intensity data were converted into HbO concentration change time series using the modified Beer-Lambert law, with differential pathlength factors set as above [38]. (4) Epoch Segmentation: HbO concentration data for the task period (35–90 s), pre-task resting baseline (5–30 s), and post-task resting period (95–150 s) were extracted for further analysis.

### Brain activation and functional connectivity analysis

Brain Activation Metric: For each channel, the average change in HbO concentration during the task period relative to the pre-task resting baseline was calculated as an indicator of task-related activation intensity for that brain region.

Functional Connectivity Analysis: Functional networks were constructed using FC_NIRS software [41]. For both the task period (35–90 s, using sliding window analysis with a 30-s window and 1-s step) and the resting periods (pre- and post-task), Pearson correlation coefficients were calculated between the HbO time series of all channel pairs. The resulting correlation coefficient matrices were subjected to Fisher’s Z-transformation (formula: Z$\:\:=\:\frac{1}{2}\mathrm{ln}\left(\frac{1+r}{1-r}\right)$, with the Z-value used as the final functional connectivity strength [42]. For resting-state analysis, global signal regression was performed on each channel’s signal beforehand.

### Statistical analysis

Data were analyzed using SPSS 24.0 software (IBM, USA). All statistical tests were two-tailed, with the significance level set at α = 0.05. Analysis of Demographic and Clinical Variables: Categorical variables are presented as frequencies (*n*), compared between groups using the *χ*² test. Continuous variables are presented as mean ± standard deviation (Mean ± SD). Data normality was assessed using the Kolmogorov-Smirnov test combined with visual inspection of Q-Q plots; homogeneity of variance was assessed using the Brown-Forsythe test. Age data were normally distributed but with unequal variances, thus the Welch test was used for overall group comparison. SDS and VAS scores in the case groups were non-normally distributed with unequal variances, compared using the Mann-Whitney *U* test. fNIRS Data Analysis: Activation levels and brain network features during task and resting states were non-normally distributed with unequal variances. Overall group comparisons were performed using the Kruskal-Wallis test. For channels showing significant overall differences, post-hoc pairwise comparisons (3 comparisons total) were conducted using the Mann-Whitney *U* test, with significance thresholds adjusted for multiple comparisons using the Bonferroni method (corrected *p* ≤ 0.0167). Mediation Effect Analysis: Bootstrap-based mediation analysis (5,000 resamples) was used to explore the mediating role of task/resting-state activation levels and brain network features in the relationship between depressive symptoms and CSP. Sex, age, and illness duration were included as covariates in the models. Significance (p-value) of the mediation effects was corrected for multiple comparisons using the Benjamini-Hochberg False Discovery Rate (FDR) method (critical *q* = 0.05). The indirect effect estimate and its 95% Bootstrap Confidence Interval (95% CI) are reported. Correlation Analysis: Spearman’s rank correlation analysis was used to explore associations between HbO signals (task/resting-state activation levels and network features) and demographic/clinical variables. Multiple comparisons were corrected using the Bonferroni method (corrected significance threshold *p* ≤ 0.01). Visualization: The BrainNet Viewer toolbox [43] and GraphPad Prism 8.0.1 software were used for image processing and result visualization.

## Results

### Analysis of demographic and clinical characteristics

A total of 129 participants were included. The case group (MDD patients) comprised 35 males (mean age 40.26 ± 17.749 years) and 51 females (mean age 39.86 ± 17.351 years). The healthy control group consisted of 19 males (mean age 45.21 ± 11.028 years) and 24 females (mean age 38.58 ± 13.351 years). Comparative analysis revealed no significant differences between the case and control groups in mean age (*p* = 0.126) or sex distribution (*p* = 0.512). Within the MDD group, the two clinical subgroups had comparable illness durations (*Z* = -0.169, *r* = 0.018, *p* = 0.866). However, significant differences in self-reported depression severity were observed using the SDS. The MDD without CSP (MDD-CSP) subgroup had significantly higher SDS scores compared to the MDD with CSP (MDD + CSP) subgroup, with a large effect size indicating a substantial difference (*Z* = -4.929, *r* = 0.53, *p* < 0.001). No missing data were encountered for fNIRS or clinical variables; all 129 participants completed all assessments and were included in the final analyses. Detailed demographic and clinical characteristics are summarized in Table 1.

**Table 1:** Demographic and clinical characteristics of participants: depressive disorder subgroups (MDD + CSP, MDD-CSP) vs. healthy controls

Group	*n*	Sex (F/ M, *n*)	Age (y, mean ± SD)	SDS (mean ± SD)	Duration of Illness (DOI, y, mean ± SD)	VAS Pain Score (mean ± SD)	Pain Intensity (*n*, None/ Mild/ Moderate/ Severe)
MMD + CSP	43	20/23	43.35 ± 16.194	61.42 ± 8.547	3.57 ± 3.106	5.58 ± 1.384	0/0/31/12
MDD-CSP	43	15/28	36.70 ± 18.127	71.40 ± 7.271	4.82 ± 5.566	0.63 ± 0.817	23/20/0/0
HC	43	19/24	41.51 ± 12.682	-	-	-	-
Statistic		1.338^△^	4.145^◎^	354.50^●***^	905.000^●^	-	-

Notes: Data presentation: Continuous variables are presented as mean ± standard deviation; categorical variables are presented as counts (*n*). Abbreviations: MDD + CSP = major depressive disorder with chronic somatic pain; MDD-CSP = major depressive disorder without chronic somatic pain; HC = healthy controls; SDS = Self-Rating Depression Scale; DOI = Duration of Illness; VAS = Visual Analogue Scale. Statistical analyses: ① Sex distribution was compared using Pearson’ s *χ*² test (^△^). ② Age was analyzed with Welch’ s ANOVA due to unequal variances (F/^◎^). ③ SDS and DOI scores (non-normally distributed) were compared between patient subgroups using the Mann–Whitney U test (U/^●^). ④ VAS and pain intensity were not statistically compared between MDD + CSP and MDD-CSP groups, as they were used as grouping criteria. Key findings: *p* < 0.001 for SDS score comparison (MDD-CSP vs. MDD + CSP). The MDD-CSP group had VAS scores < 4, so pain intensity was categorized as None or Mild only

### Differences in fNIRS activation during verbal fluency task across MDD subgroups

**Fig. 3:**
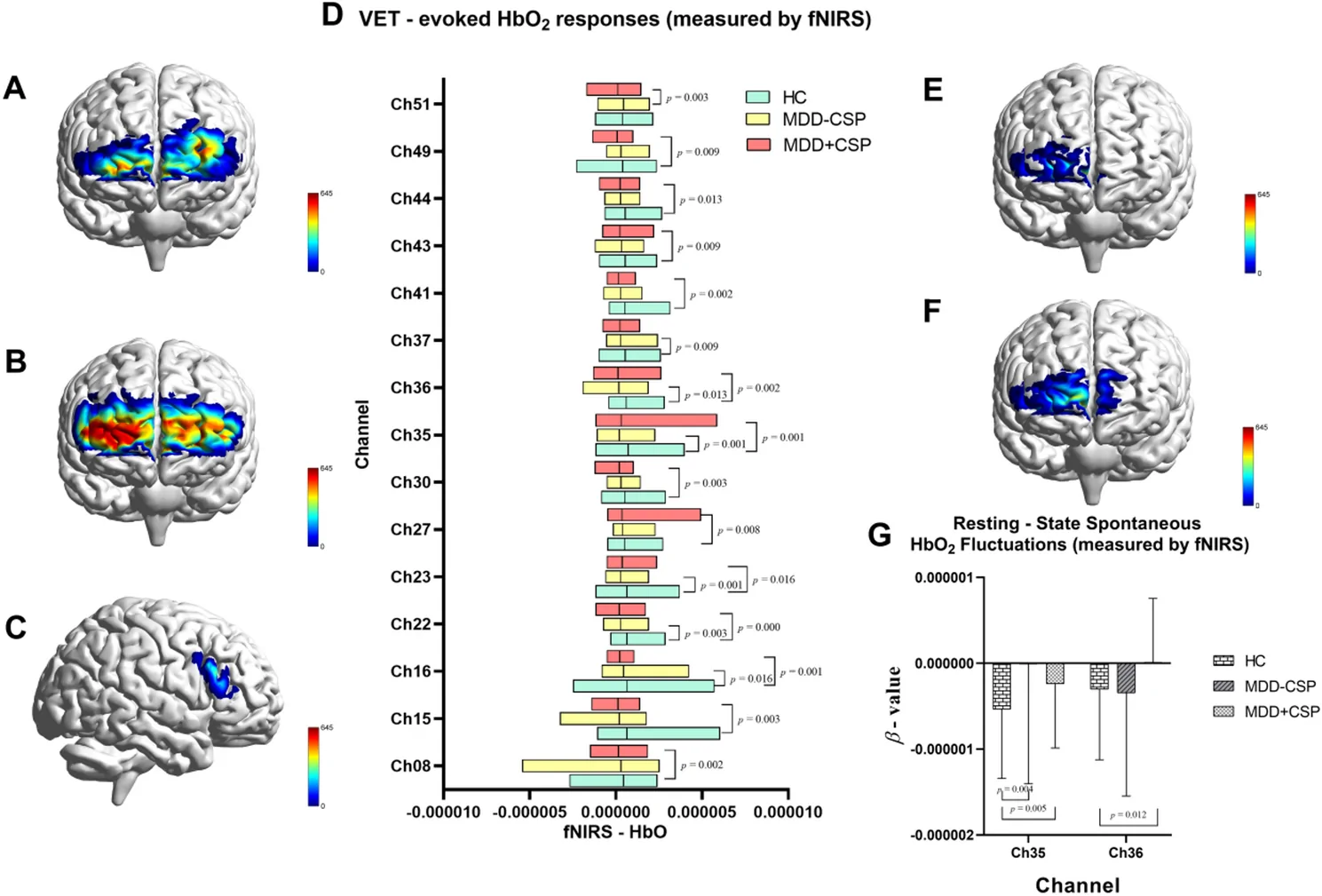
Between-group differences in cortical activation during task and resting states. (**A**–**C**) Task-state (VFT) activation comparisons: (**A**) HC vs. MDD-CSP; (**B**) HC vs. MDD + CSP; (**C**) MDD-CSP vs. MDD + CSP. Color-coded maps indicate brain regions with significant HbO activation differences (*p* ≤ 0.0167). (**D**) Quantitative analysis of task-state activation: Oxyhemoglobin (HbO) concentration changes (mean ± SD) in channels showing significant between-group differences. (**E**–**F**) Resting-state spontaneous HbO fluctuation comparisons: (**E**) HC vs. MDD-CSP; (**F**) HC vs. MDD + CSP. (**G**) Quantitative analysis of resting-state fluctuations: Amplitude of spontaneous HbO fluctuations (mean ± SD) in channels with significant between-group differences. Statistical analyses: Kruskal–Wallis test followed by post-hoc Mann–Whitney U tests with Bonferroni correction (significance threshold: *p* ≤ 0.0167). *n* = 43 for HC, *n* = 43 for MDD-CSP, *n* = 43 for MDD + CSP

**Fig. 4:**
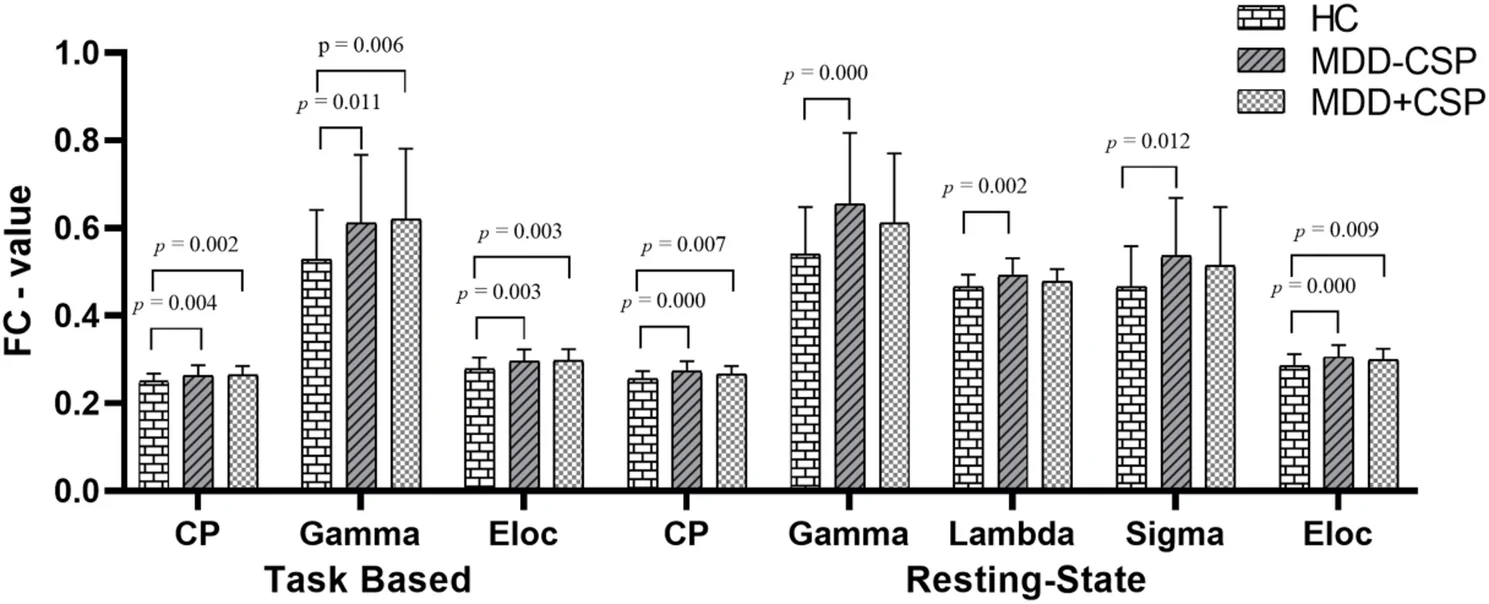
Group comparisons of task-based and resting-state functional connectivity (fNIRS). Task-state (VFT) connectivity: Bar graphs show inter-group differences in network metrics, including Connection Probability (CP), Normalized Clustering Coefficient (Gamma), and Local Efficiency (Eloc). Resting-state connectivity: Bar graphs depict inter-group differences in network metrics, including CP, Gamma, Normalized Path Length (Lambda), Small-Worldness (Sigma), and Eloc. Group definitions: HC (brick pattern), MDD-CSP (diagonal pattern), MDD + CSP (grid pattern). Statistical analyses: Kruskal–Wallis test followed by post-hoc Mann–Whitney U tests (Bonferroni correction; significance threshold: *p* ≤ 0.0167). Significant inter-group differences are annotated with exact p-values. *n* = 43 for HC, *n* = 43 for MDD-CSP, *n* = 43 for MDD + CSP

Using fNIRS, neural activity during the VFT was compared among the MDD + CSP, MDD-CSP, and HC groups, revealing abnormalities in both regional activation and network features in the patient groups. 1. Differences in Regional Activation Patterns: Overall, all MDD patients (combined MDD + CSP and MDD-CSP groups) showed widespread hypoactivation in the bilateral frontopolar (FP) region (Ch16, *χ*² = 12.246, *p* = 0.002, *η*² = 0.081; Ch22, *χ*² = 15.684, *p* < 0.001, *η*² = 0.109; Ch23, *χ*² = 11.537, *p* = 0.003, *η*² = 0.076; Ch35, *χ*² = 15.677, *p* < 0.001, *η*² = 0.109; Ch36, *χ*² = 9.032, *p* = 0.005, *η*² = 0.056). Subgroup analysis showed that the MDD-CSP group exhibited significantly reduced activation only in the right frontopolar (R-FP, Ch37) region compared to HC (*U* = 623.00, *Z* = -2.604, *r* = 0.281). In contrast, the MDD + CSP group displayed a more extensive and significant abnormal pattern: (1) Widespread hypoactivation in bilateral FP regions (Ch15, *U* = 575.50, *Z* = -3.014, *r* = 0.325; Ch27, *U* = 617.00, *Z* = -2.656, *r* = 0.286; Ch30, *U* = 574.50, *Z* = -3.023, *r* = 0.326; Ch41, *U* = 563.00, *Z* = -3.122, *r* = 0.337; Ch43, *U* = 621.50, *Z* = -2.617, *r* = 0.282); (2) Significantly reduced activation in language-related areas, including the left Broca’s area and its right-hemisphere homologue (Ch08, *U* = 568.50, *Z* = -3.075, *r* = 0.332; Ch44, *U* = 636.00, *Z* = -2.492, *r* = 0.269; Ch49, *U* = 623.00, *Z* = -2.604, *r* = 0.281); (3) Direct comparison with the MDD-CSP group showed further reduction in activation in the right-hemisphere Broca’s homologue (Ch51) (*U* = 577.50, *Z* = -2.997, *r* = 0.323). 2. Differences in Brain Network Features: At the functional brain network level, all MDD patients showed significantly increased connection probability (CP, *χ*² = 12.268, *p* = 0.002, *η*² = 0.082), normalized local clustering coefficient (Gamma, *χ*² = 9.314, *p* = 0.009, *η*² = 0.058), and local efficiency (Eloc, *χ*² = 11.981, *p* = 0.003, *η*² = 0.079) compared to HC, indicating an aberrantly heightened pattern in both information integration and local processing. In summary, comorbid chronic pain may exacerbate functional impairments in prefrontal and language-related brain regions in MDD patients. Results are visualized in Figs. 3 and 4.

### Differences in resting-state brain activity and networks across MDD subgroups based on fNIRS

This study further compared resting-state neural characteristics among the three groups (MDD + CSP, MDD-CSP, HC), with main findings as follows: (1) Differences in Regional Activation Patterns: During rest, all MDD patients overall exhibited significantly increased activation in the right frontopolar (R-FP, Ch35) region (*χ*² = 10.875, *p* = 0.004, *η*² = 0.070). Subgroup analysis showed that the MDD + CSP group had significantly higher activation levels in the right frontopolar region (Ch36) compared to HC (*U* = 632.00, *Z* = -2.526, *p* = 0.012, *r* = 0.272). (2) Differences in Brain Network Features: At the resting-state functional network level, all MDD patients showed significantly increased connection probability (CP, *χ*² = 15.567, *p* < 0.001, *η*² = 0.108) and local efficiency (Eloc, *χ*² = 14.168, *p* = 0.001, *η*² = 0.097) compared to HC. The MDD-CSP group exhibited more specific network alterations, with significantly increased normalized local clustering coefficient (Gamma, *U* = 519.00, *Z* = -3.502, *p* < 0.001, *r* = 0.378), normalized global path length (Lambda, *U* = 573.00, *Z* = -3.036, *p* = 0.002, *r* = 0.327), and small-worldness (Sigma, *U* = 633.00, *Z* = -2.518, *p* = 0.012, *r* = 0.272) compared to HC, suggesting more pronounced reorganization in global and local information processing efficiency. Detailed results are presented in Figs. 3 and 4.

### Bidirectional mediating role of right-hemisphere language area in depression-pain comorbidity

Mediation analysis (FDR-corrected) was conducted to explore the role of task/resting-state regional activation and network connectivity between chronic pain (VAS) and depressive symptoms (SDS). The main findings are as follows. During the task period, activation in the right-hemisphere Broca’s homologue (RH-Broca, Ch53) exhibited significant bidirectional mediation effects. Specifically: [1] In the “pain → brain region → depression” path, higher pain intensity (VAS) was significantly associated with reduced activation in the RH-Broca (Ch53) (*a* = -0.16, *p* < 0.001), which in turn was associated with more severe depressive symptoms (SDS, *b* = -7.54, *p* < 0.001). This mediation effect was significant (indirect effect *β* = 1.17, 95% CI: 0.66, 1.71, *p* < 0.001) [2]. In the “depression → brain region → pain” path, more severe depressive symptoms (SDS) were also associated with reduced activation in the RH-Broca (Ch53) (*a* = -0.06, *p* < 0.001), which further predicted higher pain intensity (VAS, *b* = -2.16, *p* < 0.001). This path was also significant (indirect effect *β* = 0.13, 95% CI: 0.08, 0.18, *p* < 0.001). Additionally, at an uncorrected level, other prefrontal and right-hemisphere language-related areas during the task (e.g., left frontopolar Ch16, right frontopolar Ch41/Ch43, right Broca’s homologue Ch49/Ch50) also showed potential mediation effects. However, no significant mediation effects were detected for any regional activation or whole-brain functional connectivity measures during the resting state (all *p* > 0.05).

**Fig. 5:**
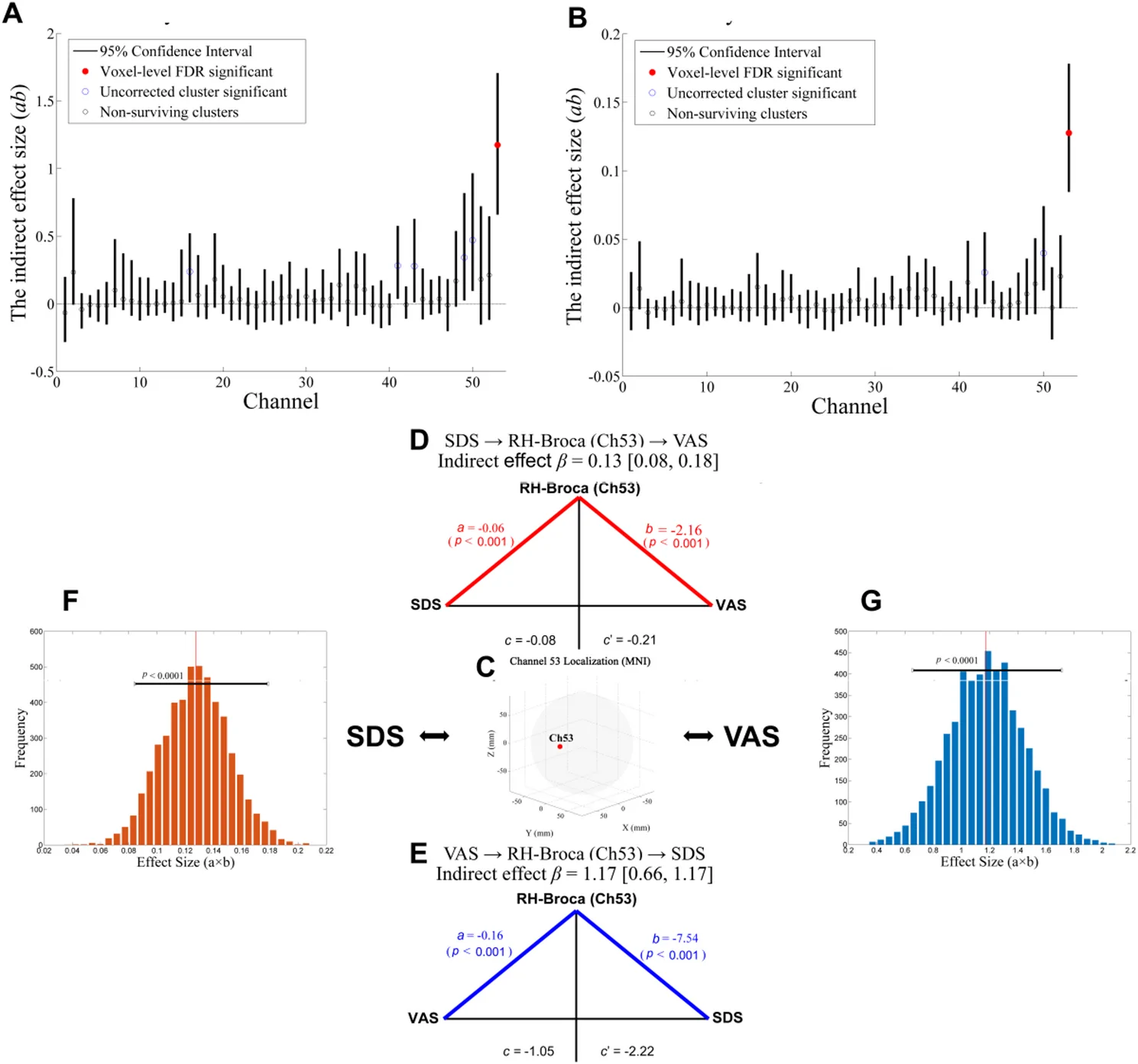
Bidirectional mediation model of the right-hemispheric Broca’s homologue (Channel 53, Ch53) linking pain intensity (Visual Analogue Scale, VAS) and depressive symptoms (Self-Rating Depression Scale, SDS). (**A**) Mediation Path 1: VAS → Ch53 activation → SDS (higher pain intensity correlates with reduced Ch53 activation, which is associated with more severe depressive symptoms). (**B**) Mediation Path 2: SDS → Ch53 activation → VAS (more severe depressive symptoms correlate with reduced Ch53 activation, which predicts higher pain intensity). (**C**) Ch53 localization: Anatomical position of the right-hemispheric Broca’s homologue (Ch53) in Montreal Neurological Institute (MNI) standard space. (**D**–**E**) Schematic mediation models: (**D**) SDS → right-hemispheric Broca’s homologue (Ch53) → VAS; (**E**) VAS → right-hemispheric Broca’s homologue (Ch53) → SDS. (**F**–**G**) Bootstrap distributions: (**F**) Bootstrap distribution of the indirect effect for the SDS→VAS path; (**G**) Bootstrap distribution of the indirect effect for the VAS→SDS path. Mediation analysis: Bootstrap-based mediation (*n* = 5,000 resamples) was performed, adjusting for covariates (sex, age, duration of illness, DOI). Significance of mediation effects was controlled via the Benjamini-Hochberg false discovery rate (FDR) method (critical value *q* = 0.05). Indirect effect estimates: VAS→Ch53→SDS (indirect effect *β* = 1.17, 95% CI: 0.66–1.71); SDS→Ch53→VAS (indirect effect *β* = 0.13, 95% CI: 0.08–0.18). *n* = 43 for HC, *n* = 43 for MDD-CSP, *n* = 43 for MDD + CSP

These results indicate that task-state functional reduction in the RH-Broca (Ch53) may play a key bidirectional mediating role between chronic pain and depressive symptoms. The effect of the path from pain to depression via this region was larger (*ab* ≈ 1.17), which suggests that for each 1-point increase in pain intensity (VAS), depressive symptoms (SDS) increase by approximately 1.17 points through suppression of RH-Broca, indicating a clinically relevant mediating pathway. The path from depression to pain via this region was smaller (*ab* ≈ 0.13), hinting that depressive mood may also slightly amplify pain perception. This finding highlights the central role of task-specific brain activity in the comorbidity mechanism of pain and depression. The detailed path model is shown in Fig. 5.

### Correlation analysis between fNIRS-HbO and demographic/clinical variables

Spearman correlation analysis was used to examine associations between fNIRS-HbO signals and various variables, revealing distinct association patterns during task and resting states. (1) During the VFT, demographic and clinical variables showed significant correlations with activation in specific brain regions. Regarding sex, female participants showed reduced activation in Broca’s area (Ch03, *p* = 0.005), but stronger activation in the left frontal eye field (Ch24, *p* = 0.005). Older age was associated with reduced activation in the left dorsolateral prefrontal cortex (Ch14, *p* = 0.008). For clinical variables, longer illness duration correlated with reduced activation in bilateral dorsolateral prefrontal regions (Ch17, *p* = 0.003; Ch34, *p* = 0.004). Higher pain intensity (VAS) was associated with more widespread activation decreases, primarily involving Broca’s area/ its right-hemisphere homologue (e.g., Ch02, *p* = 0.002; Ch49, *p* = 0.006; Ch50, *p* < 0.001; Ch51, *p* < 0.001; Ch53, *p* < 0.001) and right prefrontal regions (Ch41, *p* = 0.002; Ch43, *p* = 0.005). Furthermore, more severe depressive symptoms (SDS) were generally correlated with reduced activation in bilateral supplementary/premotor areas (e.g., Ch01, *p* = 0.007; Ch47, *p* = 0.006; Ch52, *p* = 0.001), the right prefrontal cortex (Ch41, *p* = 0.010), and the right-hemisphere Broca’s homologue (Ch50, *p* = 0.007; Ch53, *p* < 0.001). (2) During the resting state, the correlation pattern differed. Sex differences manifested as significantly lower activation in females compared to males in bilateral prefrontal regions (e.g., Ch19, *p* = 0.007; Ch48, *p* = 0.004) and Broca’s area (Ch02, *p* < 0.001). Conversely, older age was associated with increased activation in bilateral prefrontal regions (Ch19, *p* = 0.006; Ch36, *p* = 0.004). These results reveal distinct characteristics of brain function associations during task and resting states. Detailed data are presented in Table 2.

**Table 2:** Correlations of demographic and clinical variables with fNIRS-HbO signals during verbal fluency task and resting state

Experimental States	Channel (Brain Region)	Sex	Age (*y*)	DOI (*y*)	VAS	SDS
Task - Based	Ch01 (SMA + PMC)	0.050	-0.031	-0.138	-0.048	-0.290^*^
	Ch02 (BA)	-0.006	-0.101	-0.074	-0.326^**^	-0.217
	Ch03 (BA)	-0.246^*^	-0.103	-0.128	0.165	-0.130
	Ch14 (DLPFC)	-0.026	-0.232^*^	-0.254	0.103	-0.091
	Ch17 (DLPFC)	-0.017	-0.059	-0.313^*^	-0.158	-0.107
	Ch24 (FEF)	0.248^*^	-0.053	0.141	-0.014	-0.154
	Ch34 (DLPFC)	-0.109	-0.151	-0.305^**^	-0.149	-0.159
	Ch41 (FP)	-0.072	-0.133	-0.115	-0.326^**^	-0.275^*^
	Ch43 (FP)	0.002	-0.107	-0.184	-0.298^*^	-0.214
	Ch47 (SMA + PMC)	-0.064	0.003	0.000	-0.067	-0.292^*^
	Ch49 (BA)	0.017	-0.073	-0.116	-0.295^*^	-0.156
	Ch50 (BA)	-0.158	-0.048	-0.002	-0.374^***^	-0.291^*^
	Ch51 (BA)	0.073	-0.094	-0.063	-0.390^***^	0.049
	Ch52 (SMA + PMC)	-0.096	-0.051	-0.125	-0.069	-0.344^**^
	Ch53 (BA)	-0.044	-0.077	-0.047	-0.481^***^	-0.538^***^
Resting State	Ch02 (BA)	-0.329^***^	-0.006	-0.122	0.080	-0.109
	Ch19 (FP)	-0.235^*^	0.239^*^	-0.012	-0.042	-0.063
	Ch36 (FP)	-0.176	0.254^**^	0.125	0.138	-0.079
	Ch48 (FP)	-0.249^**^	0.013	-0.173	-0.029	-0.131

Notes: Data are Spearman’s rank correlation coefficients (*ρ*). Sample sizes: [1] Correlations with demographic variables included all participants (*n* = 129); [2] Correlations with clinical variables included only patients (MDD-CSP + MDD+CSP, *n* = 86). Abbreviations: VAS = Visual Analogue Scale; SDS = Self-Rating Depression Scale; DOI = duration of illness; HC = healthy controls; MDD + CSP = major depressive disorder with chronic somatic pain; MDD-CSP = major depressive disorder without chronic somatic pain; FP = frontal pole; FEF = frontal eye fields; DLPFC = dorsolateral prefrontal cortex; BA = Broca’s area; SMA/PMC = supplementary motor area/premotor cortex. Statistical significance: Bonferroni-corrected threshold (*p* < 0.010). * *p* < 0.010, ** *p* < 0.005, *** *p* < 0.001. Channel correspondence: ChXX labels refer to the brain regions illustrated in Fig. 2

## Discussion

This case-control fNIRS study systematically explored the neural substrates underlying MDD comorbid with CSP. Our key findings identified two distinct neural alterations: widespread frontopolar dysfunction as a transdiagnostic feature of MDD, and critically, chronic somatic pain (CSP)-specific suppression of RH-Broca. Mediation analysis further confirmed that RH-Broca serves as a central hub mediating the bidirectional pain-depression loop. These results not only advance the mechanistic understanding of MDD-CSP comorbidity beyond simple symptom overlap but also provide compelling neuroimaging evidence for classifying “pain-comorbid MDD” as a circuit-defined subtype. Importantly, this work offers direct implications for developing targeted neuromodulatory interventions, aligning with the journal’s focus on translational psychiatric research.

### Frontopolar dysfunction in MDD: a core feature with task-rest dissociation

Consistent with extensive prior literature [27, 44], our study confirms that bilateral frontopolar (FP) hypoactivation during cognitive effort is a transdiagnostic feature of MDD. The FP is crucial for integrative executive functions and metacognition; its dysfunction likely contributes to the impaired cognitive control and emotion regulation central to depression [45]. Notably, we observed a task-rest dissociation: while hypoactive during the Verbal Fluency Task (VFT), the right FP exhibited hyperactivity at rest. This pattern may reflect a failure in the dynamic suppression of the default mode network (DMN) [46], potentially linked to maladaptive rumination. Importantly, the MDD + CSP group showed more extensive FP hypoactivation, suggesting that chronic pain acts as an additional cognitive load, further depleting the already compromised prefrontal resources.

### Identifying a potential “pain-comorbid” MDD subtype: the pivotal role of the right-hemispheric broca’s homologue

A core and novel finding of this study is the CSP-specific suppression of RH-Broca (i.e., the right inferior frontal gyrus, rIFG). This region extends beyond classical language function, serving as a hub for integrating affective, sensory, and cognitive information within the salience network.

Mechanistic Model for Subtyping: The co-occurrence of “right FP resting hyperactivity” and “RH-Broca task suppression” provides a neural signature that may help delineate a “pain-comorbid” MDD subtype. We propose a dual dysfunction model: FP hyperactivity may sustain pain-related catastrophizing and negative self-referential thought, while RH-Broca suppression impairs the cognitive-emotional integration necessary for adaptive pain coping and mood regulation. This “resource depletion and dysregulation” model contrasts with putative “pure” MDD subtypes, which may involve more isolated dysfunction within classic mood-regulation circuits (e.g., dorsolateral prefrontal-amygdala). This notion of distinct yet interacting neural pathways for pain and comorbid depression is supported by recent preclinical evidence [20]. This is supported by findings that attenuated hemodynamic responses in prefrontal regions including the frontal pole and Broca’s area are correlated with depression severity and mediate its relationship with psychosocial factors, underscoring their biomarker potential [47, 48].

Bidirectional Mediation and Clinical Phenotype: The bidirectional mediation effect through RH-Broca statistically formalizes this pain-depression interaction. Shared genetic underpinnings may predispose individuals to both chronic pain and internalizing psychopathology like depression [49, 50], providing a basis for the high comorbidity. The stronger path from pain to depression (*β* = 1.17) aligns with a “pain-driven depression” phenotype, where chronic nociception directly dampens rIFG function. The reverse, weaker path (*β* = 0.13) suggests depressive states may exacerbate pain perception partly through this same circuit. This neural mechanism may underlie the clinical observation that patients with comorbid pain often present with more somatic symptoms, which can “dilute” classic affective complaints and potentially lead to underestimation of depression severity on standard scales.

### Brain network reorganization: from compensatory effort to maladaptive rigidity

At the network level, our findings reveal a dynamic trajectory that may have implications for disease progression and treatment resistance.

Task-State Compensation: The increased local clustering and efficiency (Gamma, Eloc) during the VFT in all MDD patients likely represents a compensatory mechanism. Faced with hypoactivation of key hubs (FP, RH-Broca), the brain may strengthen local processing within sub-networks to maintain baseline task performance.

Resting-State Maladaptation in MDD-CSP: The significantly elevated small-worldness (Sigma) in the MDD-CSP group at rest is particularly telling. While a small-world topology is normally efficient, an excessively high Sigma indicates a shift toward network rigidity—high local clustering at the expense of global integration. This “hyper-optimized but inflexible” state is increasingly linked to poor treatment outcomes and cognitive inflexibility in depression [46, 51], and aligns with the reported imbalance between network integration and segregation in chronic pain conditions [52]. Critically, such aberrant baseline network properties have been demonstrated to directly predict poorer responsiveness to psychological therapies in patients with comorbid chronic pain and depression [32], underscoring the clinical relevance of this maladaptive reorganization.

Network Modulation by Chronic Pain [53]: The absence of this high-Sigma signature in the MDD + CSP group is intriguing. This aligns with findings that comorbid depression in chronic pain conditions is associated with reduced small-worldness, suggests that the pathophysiology of chronic pain does not merely add to depressive network changes but may actively reshape them into a different abnormal configuration, characterized more by RH-Broca suppression and resource competition than by global network rigidification. This further supports the distinctiveness of the comorbid subtype from a network neuroscience perspective.

### Clinical translation: toward circuit-targeted and fnirs-guided personalized therapy our findings directly translate into a roadmap for personalized intervention

Defining the Target Population: Neurostimulation therapies like repetitive transcranial magnetic stimulation (rTMS) targeting the rIFG (RH-Broca) hold particular promise for the “pain-comorbid” MDD subgroup. This is especially relevant for patients with treatment-resistant depression where chronic pain, particularly musculoskeletal pain like low back pain, is a prominent feature and has been associated with attenuated response to standard rTMS protocols [54]. Our model provides a neural rationale for this clinical observation.

fNIRS as a Translational Tool: The portability and tolerance of fNIRS position it uniquely for clinical translation. It can evolve from a research tool into a “biomarker navigation system” for personalized neuromodulation: (1) Baseline Stratification: The VFT can be used to identify patients with the RH-Broca suppression phenotype. (2) Target Engagement Verification: Real-time fNIRS can monitor hemodynamic changes at the stimulation site during rTMS sessions. (3) Treatment Response Tracking: Serial fNIRS assessments can objectively track the normalization of the FP and RH-Broca circuit, providing a neural correlate of recovery beyond subjective scales. This approach aligns with the future vision of connectivity-guided therapy [55].

### Limitations and future directions

This study has several limitations. First, its cross-sectional nature precludes causal inference from the mediation models; longitudinal studies are needed. Second, fNIRS has limited sensitivity to subcortical and deep cortical structures involved in pain and affect (e.g., insula, anterior cingulate). Future multimodal imaging combining fNIRS with fMRI or EEG is warranted. Third, while we controlled for medication washout, long-term pharmacological history may have enduring effects on neuroplasticity. Fourth, the heterogeneity of chronic pain (neuropathic vs. nociceptive) was not accounted for, which future studies should address to refine mechanistic understanding. Finally, our promising translational framework requires direct validation in interventional trials coupling fNIRS biomarker assessment with targeted neuromodulation.

## Conclusion

In conclusion, this fNIRS study delineates distinct neural circuit alterations associated with MDD comorbid with chronic pain, centered on the dysfunctional interplay between the frontopolar cortex and the right-hemispheric Broca’s homologue. These findings advance the mechanistic understanding of pain-depression comorbidity beyond simple comorbidity models, supporting the notion of a neural circuit-defined subtype. They provide a solid empirical foundation for developing biologically informed diagnostics and launching a new era of personalized, circuit-targeted neuromodulation therapies.

## Supplementary Information

Below is the link to the electronic supplementary material.

Supplementary Material 1

Supplementary Material 2

Supplementary Material 3

Supplementary Material 4

Supplementary Material 5

Supplementary Material 6

## Data Availability

The datasets generated and analyzed during this study are not publicly available but are available from the corresponding author upon reasonable request.

## Abbreviations

BA: Broca’s area
Ch53: Channel 53
CP: Connection Probability
DLPFC: Dorsolateral prefrontal cortex
DOI: Duration of illness
Eloc: Local Efficiency
FEF: Frontal eye fields
FP: Frontal pole
fNIRS: Functional near-infrared spectroscopy
Gamma: Normalized Clustering Coefficient
HC: Healthy controls
Lambda: Normalized Path Length
MDD + CSP: Major depressive disorder with chronic somatic pain
MDD-CSP: Major depressive disorder without chronic somatic pain
RH-Broca: Right-hemispheric Broca’s homologue
SDS: Self-Rating Depression Scale
SMA/PMC: Supplementary motor area/premotor cortex
Sigma: Small-Worldness
SNR: Signal-to-noise ratio
VAS: Visual analogue scale
DSM-5: Diagnostic and Statistical Manual of Mental Disorders, 5th Edition
