# Supplementary material for: Right broca homologue mediates the pain-depression circuit: a case-control Functional Near-Infrared Spectroscopy (fNIRS) study on language network remodeling in chronic pain-depression comorbidity*
Source: BMC Psychiatry. 2026 Jun 23;26:741. doi: 10.1186/s12888-026-08323-3 (PMC13595546; doi:10.1186/s12888-026-08323-3)
Supplement: Supplementary file 1 [file 12888_2026_8323_MOESM1_ESM.docx]

Fig. 1 Flow diagram of participant recruitment, eligibility screening, and group allocation. Abbreviations: MDD, major depressive disorder; CSP, chronic somatic pain; VAS, visual analog scale; SCID-I/NP, Structured Clinical Interview for DSM-IV Axis I Disorders Non-Patient Edition; fNIRS, functional near-infrared spectroscopy.
