# Supplementary material for: Right broca homologue mediates the pain-depression circuit: a case-control Functional Near-Infrared Spectroscopy (fNIRS) study on language network remodeling in chronic pain-depression comorbidity*
Source: BMC Psychiatry. 2026 Jun 23;26:741. doi: 10.1186/s12888-026-08323-3 (PMC13595546; doi:10.1186/s12888-026-08323-3)
Supplement: Supplementary file 2 [file 12888_2026_8323_MOESM2_ESM.docx]

Fig. 2. fNIRS channel configuration and Verbal Fluency Task (VFT) paradigm. (A) A 16-source, 16-detector optode array with 3 cm source-detector separation, forming 53 channels, covering the prefrontal and language-related cortices (FP: frontal pole; FEF: frontal eye fields; DLPFC: dorsolateral prefrontal cortex; BA: Broca’s area; SMA+PMC: supplementary motor area + premotor cortex). (B) A 150-second block design paradigm: 30-second baseline rest (counting 1–16), 60-second task period (generating words from high-frequency Chinese characters), and 60-second post-task recovery (counting 1–32). *n* = 43 per group.
