# Supplementary material for: Right broca homologue mediates the pain-depression circuit: a case-control Functional Near-Infrared Spectroscopy (fNIRS) study on language network remodeling in chronic pain-depression comorbidity*
Source: BMC Psychiatry. 2026 Jun 23;26:741. doi: 10.1186/s12888-026-08323-3 (PMC13595546; doi:10.1186/s12888-026-08323-3)
Supplement: Supplementary file 3 [file 12888_2026_8323_MOESM3_ESM.docx]

Fig. 3. Between-group differences in cortical activation during task and resting states. (A–C) Task-state (VFT) activation comparisons: (A) HC vs. MDD-CSP; (B) HC vs. MDD+CSP; (C) MDD-CSP vs. MDD+CSP. Color-coded maps indicate brain regions with significant HbO activation differences (p ≤ 0.0167). (D) Quantitative analysis of task-state activation: Oxyhemoglobin (HbO) concentration changes (mean ± SD) in channels showing significant between-group differences. (E–F) Resting-state spontaneous HbO fluctuation comparisons: (E) HC vs. MDD-CSP; (F) HC vs. MDD+CSP. (G) Quantitative analysis of resting-state fluctuations: Amplitude of spontaneous HbO fluctuations (mean ± SD) in channels with significant between-group differences. Statistical analyses: Kruskal–Wallis test followed by post-hoc Mann–Whitney U tests with Bonferroni correction (significance threshold: *p* ≤ 0.0167). *n* = 43 for HC, *n* = 43 for MDD-CSP, *n* = 43 for MDD+CSP.
