# Supplementary material for: Right broca homologue mediates the pain-depression circuit: a case-control Functional Near-Infrared Spectroscopy (fNIRS) study on language network remodeling in chronic pain-depression comorbidity*
Source: BMC Psychiatry. 2026 Jun 23;26:741. doi: 10.1186/s12888-026-08323-3 (PMC13595546; doi:10.1186/s12888-026-08323-3)
Supplement: Supplementary file 4 [file 12888_2026_8323_MOESM4_ESM.docx]

Fig. 5. Bidirectional mediation model of the right-hemispheric Broca’s homologue (Channel 53, Ch53) linking pain intensity (Visual Analogue Scale, VAS) and depressive symptoms (Self-Rating Depression Scale, SDS). (A) Mediation Path 1: VAS → Ch53 activation → SDS (higher pain intensity correlates with reduced Ch53 activation, which is associated with more severe depressive symptoms).

(B) Mediation Path 2: SDS → Ch53 activation → VAS (more severe depressive symptoms correlate with reduced Ch53 activation, which predicts higher pain intensity). (C) Ch53 localization: Anatomical position of the right-hemispheric Broca’s homologue (Ch53) in Montreal Neurological Institute (MNI) standard space. (D–E) Schematic mediation models: (D) SDS → right-hemispheric Broca’s homologue (Ch53) → VAS; (E) VAS → right-hemispheric Broca’s homologue (Ch53) → SDS. (F–G) Bootstrap distributions: (F) Bootstrap distribution of the indirect effect for the SDS→VAS path; (G) Bootstrap distribution of the indirect effect for the VAS→SDS path. Mediation analysis: Bootstrap-based mediation (*n* = 5,000 resamples) was performed, adjusting for covariates (sex, age, duration of illness, DOI). Significance of mediation effects was controlled via the Benjamini-Hochberg false discovery rate (FDR) method (critical value *q* = 0.05). Indirect effect estimates: VAS→Ch53→SDS (indirect effect *β* = 1.17, 95% CI: 0.66–1.71); SDS→Ch53→VAS (indirect effect *β* = 0.13, 95% CI: 0.08–0.18). *n* = 43 for HC, *n* = 43 for MDD-CSP, *n* = 43 for MDD+CSP.
