# Supplementary material for: Right broca homologue mediates the pain-depression circuit: a case-control Functional Near-Infrared Spectroscopy (fNIRS) study on language network remodeling in chronic pain-depression comorbidity*
Source: BMC Psychiatry. 2026 Jun 23;26:741. doi: 10.1186/s12888-026-08323-3 (PMC13595546; doi:10.1186/s12888-026-08323-3)
Supplement: Supplementary file 5 [file 12888_2026_8323_MOESM5_ESM.docx]

Fig. 4. Group comparisons of task-based and resting-state functional connectivity (fNIRS). Task-state (VFT) connectivity: Bar graphs show inter-group differences in network metrics, including Connection Probability (CP), Normalized Clustering Coefficient (Gamma), and Local Efficiency (Eloc). Resting-state connectivity: Bar graphs depict inter-group differences in network metrics, including CP, Gamma, Normalized Path Length (Lambda), Small-Worldness (Sigma), and Eloc. Group definitions: HC (brick pattern), MDD-CSP (diagonal pattern), MDD+CSP (grid pattern). Statistical analyses: Kruskal–Wallis test followed by post-hoc Mann–Whitney U tests (Bonferroni correction; significance threshold: *p* ≤ 0.0167). Significant inter-group differences are annotated with exact p-values. *n* = 43 for HC, *n* = 43 for MDD-CSP, *n* = 43 for MDD+CSP.
